# Impact of early intervention with onabotulinumtoxinA treatment in adult patients with post-stroke lower limb spasticity: results from the double-blind, placebo-controlled, phase 3 REFLEX study

**DOI:** 10.1007/s00702-020-02251-6

**Published:** 2020-10-27

**Authors:** Atul T. Patel, Anthony B. Ward, Carolyn Geis, Wolfgang H. Jost, Chengcheng Liu, Rozalina Dimitrova

**Affiliations:** 1 Kansas City Bone and Joint Clinic, Overland Park, KS USA; 2 Faculty of Health and North Staffordshire Rehabilitation Centre, Haywood Hospital, Staffordshire University, Stoke on Trent, UK; 3 Brooks Rehabilitation/Physician Group, Halifax Health, Daytona Beach, FL USA; 4 Department of Neurology, University of Freiburg, Freiburg, Baden-Württemberg Germany; 5 Parkinson-Klinik Ortenau GmbH & Co KG, Kreuzbergstr. 12-16, 77709 Wolfach, Germany; 6 Allergan Plc, Madison, NJ USA; 7 Allergan Plc, Irvine, CA USA

**Keywords:** Spasticity, Stroke, OnabotulinumtoxinA, Early intervention

## Abstract

The aim of this study in patients with post-stroke lower limb spasticity (PSLLS) was to evaluate the relationship between time of onabotulinumtoxinA treatment relative to stroke and efficacy outcomes. This was a phase 3, international, multicenter, randomized, 12-week, double-blind study, followed by a repeated treatment, open-label extension. Patients were aged 18–85 years with PSLLS (Modified Ashworth Scale [MAS] ≥ 3) of the ankle with the most recent stroke occurring ≥ 3 months before screening. Patients (double-blind phase) were randomized (*n* = 468) to onabotulinumtoxinA 300–400 U (300 U, mandatory ankle muscles (gastrocnemius, soleus, tibialis posterior); and ≤ 100 U, optional lower limb muscles (flexor digitorum longus, flexor hallucis longus, flexor digitorum brevis, extensor hallucis, and rectus femoris]) or placebo. Primary endpoint: MAS change from baseline (average score of weeks 4 and 6). Secondary endpoints: physician-assessed Clinical Global Impression of Change (CGI) average score of weeks 4 and 6 and physician-assessed Goal Attainment Scale (GAS; active and passive, weeks 8 and 12). When stratified by time since stroke (≤ 24 months, *n* = 153; > 24 months, *n* = 315, post hoc), patients treated ≤ 24 months post-stroke experienced greater improvements from baseline versus placebo in MAS (− 0.31 vs − 0.17), CGI (0.49 vs 0.12), and passive GAS scores (week 12, 0.37 vs 0.26). A ≥  − 1-point improvement in active (week 12; *p* = 0.04) and passive (week 8; *p* = 0.02) GAS scores versus placebo was achieved by more patients treated ≤ 24 months post-stroke; in patients treated > 24 months post-stroke, improvements were only observed in active scores (week 8; *p* = 0.04). OnabotulinumtoxinA 300–400 U was well tolerated, with no new safety findings.

**Electronic supplementary material:**

The online version of this article (10.1007/s00702-020-02251-6) contains supplementary material, which is available to authorized users.

## Introduction

Post-stroke complications are multifaceted and include spasticity of the upper and lower limbs. Post-stroke spasticity (PSS), generally defined as a motor disorder associated with hypertonicity and hyperexcitable reflexes (Lance 1980), can present as early as 1 week after stroke (Lundström et al. 2010; Sommerfeld et al. 2004). PSS has a wide prevalence rate in the literature, ranging from approximately 4–66% (Leathley et al. 2004; Martin et al. 2014; Urban et al. 2010; Wissel et al. 2013, 2010), which is partly attributed to variations in measures of assessment used to define PSS; for example, Modified Ashworth Scale (MAS) score > 0 or ≥ 1 or Tone Assessment Scale score > 0 (Wissel et al. 2013). Complications associated with spasticity, especially among patients with lower limb spasticity, include impairment of mobility and motor function (Martin et al. 2014), the development of contractures (O'Dwyer et al. 1996), and reduced quality of life (Doan et al. 2012; Urban et al. 2010).

Management of PSS includes physical modalities (e.g., stretching, range-of-motion exercises, ultrasound) (Duncan et al. 2005; Francisco and McGuire 2012) and pharmacologic therapies (e.g., including intrathecal and oral baclofen, gabapentin, dantrolene, and botulinum toxins) (Duncan et al. 2005; Thibaut et al. 2013). OnabotulinumtoxinA (BOTOX^®^; Allergan plc, Dublin, Ireland) is one type of botulinum toxin approved for the treatment of upper limb spasticity in the United States and most regions worldwide. On the basis of an international development program, including the REFLEX Study (Wein et al. 2018), onabotulinumtoxinA was approved by the US Food and Drug Administration (Allergan plc 2016) for the treatment of lower limb spasticity in adults and is marketed worldwide (including Australia, Canada, Europe, Japan, and New Zealand) (Adis R&D Insight).

The clinical efficacy and safety of onabotulinumtoxinA in patients with post-stroke lower limb spasticity (PSLLS) have been observed in a multicenter, randomized, double-blind, placebo-controlled trial of 120 Japanese patients who received 300 U of onabotulinumtoxinA into the medial and lateral head of the gastrocnemius, soleus, and tibialis posterior muscles (Kaji et al. 2010). In the REFLEX Study—a large international, phase 3, multicenter, placebo-controlled study—onabotulinumtoxinA reduced muscle tone and spasticity of the ankle during the 12-week, double-blind phase (Wein et al. 2018). Improvements in ankle spasticity, as measured by the MAS, the Clinical Global Impression of change (CGI), and the Goal Attainment Scale (GAS), were significantly greater for patients treated with onabotulinumtoxinA compared with placebo. In the 1-year open-label extension of the study, sustained benefit of up to three repeated treatments of onabotulinumtoxinA was observed in patients with PSLLS of the ankle. No new safety signals were observed.

Little is known regarding the relationship between the time of onabotulinumtoxinA treatment initiation post-stroke and clinical outcomes among patients with PSLLS. Preliminary evidence among a cohort of patients with mixed upper or lower limb spasticity supports the benefit of early treatment of PSS (Wissel et al. 2010). In the primary REFLEX Study, the subgroup of patients with time since stroke ≤ 48 months consistently demonstrated more favorable efficacy outcomes after onabotulinumtoxinA therapy than patients with time since stroke > 48 months (data on file), suggesting that time since stroke was an important factor in treatment response. To further investigate this finding, we conducted a secondary analysis of this prospective analysis among patients stratified by time of treatment initiation (i.e., ≤ 24 or > 24 months) since stroke to evaluate outcome measures (including MAS, CGI, GAS, and speed of gait).

## Methods

### Study design

**Fig. 1:**
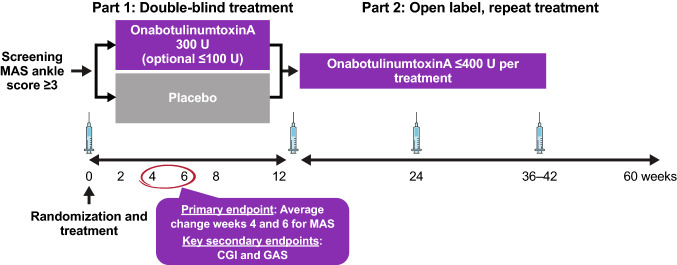
Study design. Modified Ashworth Scale. *CGI* clinical global impression of change, *GAS* Goal Attainment Scale, *MAS* Modified Ashworth Scale

This was a global, multicenter, randomized, double-blind, placebo-controlled phase 3 study of onabotulinumtoxinA for the treatment of PSLLS followed by a repeated-treatment, open-label extension (Fig. 1). A more comprehensive description of the REFLEX study design has been published previously (Wein et al. 2018). This secondary analysis focuses on findings from the double-blind phase with respect to the impact of time to treatment initiation of onabotulinumtoxinA on efficacy outcomes in patients with PSLLS.

The REFLEX study (ClinicalTrials.gov Identifier NCT01575054) was conducted at 60 sites throughout Canada, the United States, Czech Republic, Germany, Hungary, Poland, Russia, the United Kingdom, and South Korea. All investigators obtained appropriate institutional review board or independent ethics committee approval before study initiation, and the study was conducted in accordance with International Council for Harmonisation Good Clinical Practice. Written informed consent was obtained from each patient before study enrollment.

### Study treatment, doses, and injection sites

OnabotulinumtoxinA or placebo (0.9 mg sodium chloride) was reconstituted with sterile saline (4 mL of preservative-free 0.9% normal saline to each 100 U). Patients received intramuscular injections of onabotulinumtoxinA 300 U or placebo into three sites each of the gastrocnemius (medial and lateral heads), soleus, and tibialis posterior muscles (i.e., mandatory ankle muscles; Table 1). An optional dose of up to 100 U onabotulinumtoxinA or placebo was injected into the flexor digitorum longus, flexor digitorum brevis, flexor hallucis longus, extensor hallucis, or rectus femoris if clinically indicated. The need to inject the rectus femoris was determined by a clinical evaluation and a MAS knee score of ≥ 1. The need to inject the remaining optional muscles was based on the investigator’s clinical judgment.

**Table 1:** Mandatory and optional doses and muscles for injection

	Dose
Mandatory muscles	
Gastrocnemius medial head	75 U (25 U × 3 sites)
Gastrocnemius lateral head	75 U (25 U × 3 sites)
Soleus	75 U (25 U × 3 sites)
Tibialis posterior	75 U (25 U × 3 sites)
Total dose	300 U
Optional muscles	
Flexor digitorum longus	50 U (25 U × 2 sites)
Flexor digitorum brevis	25 U (1 site)
Flexor hallucis longus	50 U (25 U × 2 sites)
Extensor hallucis	25 U (1 site)
Rectus femoris	100 U (25 U × 4 sites)
Total dose	≤ 100 U

Dilution: 4 mL of preservative-free 0.9% normal saline to each 100 U

The injector and patient were blinded to whether active drug or placebo was given. Study treatments were provided in identical vials and cartons to maintain masking of the study treatment. To ensure that the injector remained blinded in the double-blind treatment phase, an independent drug reconstitutor was responsible for preparing the study medication according to the specific dilution requirements.

### Patient selection

The study enrolled men and women aged 18–85 years with a diagnosis of PSLLS (determined by a MAS score ≥ 3 in the ankle plantar flexors), with the most recent stroke occurring ≥ 3 months before screening. Enrolled patients were either naive to onabotulinumtoxinA or, if previously treated, had undergone no treatment with onabotulinumtoxinA for ≥ 20 weeks (spasticity indication) or ≥ 12 weeks (any other indication) before the screening visit.

Patients were excluded from study participation if there was an etiology other than stroke contributing to spasticity or if they had spasticity in the contralateral leg requiring treatment, if there was any medical or neurologic condition that might put the patient at increased risk with exposure to onabotulinumtoxinA, or if the patient had an intrathecal baclofen pump. Women of childbearing potential who were not using a reliable method of contraception or women who were pregnant, nursing, or planning a pregnancy during the study period were also excluded.

During the double-blind phase, the initiation of any medications for spasticity, muscle relaxants, or antiepileptic medications was prohibited. Only those on a stable dose and regimen before the first day of the study were permitted. The initiation of physical therapy or the use of static or dynamic splints within 14 days of the first study visit was also prohibited. Patients who entered the study receiving any of the aforementioned treatments were to remain on a stable dose or regimen throughout the double-blind phase.

### Assessments

#### Efficacy

The primary efficacy measure was the MAS of the ankle, which was assessed at baseline and at weeks 2, 4, 6, 8, and 12. Scores of 0, 1, 1 +, 2, 3, or 4 were coded as 0, 1, 2, 3, 4, or 5, respectively. Key secondary efficacy measures included CGI by physician and GAS by physician and patient (active and passive goals). The CGI, a 9-point scale ranging from − 4 (very marked worsening) to 4 (very marked improvement), was assessed at weeks 2, 4, 6, 8, and 12. The GAS is a 6-point scale ranging from − 3 (worse than start) to 2 (much more than expected) and was assessed at weeks 8 and 12. Speed of gait (defined as the time in seconds that it took the patient to walk 10 m) was assessed at baseline and at weeks 6 and 12.

#### Safety

Safety assessments included a physical examination (baseline), clinical laboratory evaluation (week 12), and vital signs (baseline and week 12). Adverse events (AEs) including severity and relation to study drug were recorded at baseline and at each follow-up visit.

### Outcomes

The primary outcome was the MAS of the ankle change from baseline to the average score of weeks 4 and 6. Secondary outcomes included the physician-assessed CGI average score of weeks 4 and 6 and the physician- and patient-assessed GAS (active and passive) at weeks 8 and 12. Assessment of the GAS scores was defined based on the absolute GAS scores, the proportion of patients who achieved active or passive goals (GAS score ≥ 0), and the proportion of patients who made progress toward active or passive goals (GAS score ≥  − 1). Other outcomes included the change from baseline in speed of gait at weeks 6 and 12.

### Statistical analysis

The double-blind phase of the study randomized patients in a 1:1 ratio through an interactive voice-response or web-response system to receive a single injection of onabotulinumtoxinA or placebo. All efficacy, baseline, and demographic analyses were performed on the intent-to-treat (ITT) population, defined as all patients who were randomized, regardless of actual treatment received. Safety analyses were performed on the safety population, defined as all patients who received ≥ 1 treatment.

The primary comparison between treatment groups was conducted by analysis of covariance (ANCOVA) at a significance level of 0.05. ANCOVA was performed with treatment and study center as factors, with ankle MAS at baseline, and muscles injected as covariates. To address missing data at both weeks 4 and 6, the imputation was done sequentially by the patient’s previously observed score, multiplied by the ratio of the within-group mean score at that visit, divided by the within-group mean score at the previous visit. For all secondary outcomes except CGI by physician, observed data without imputation were analyzed using ANCOVA, with treatment center as a factor, and baseline ankle MAS and muscles injected as covariates. For CGI by physician, descriptive statistics were presented by treatment group and analyzed using ANCOVA, with baseline MAS, muscles injected, treatment, and investigator site as covariates. Efficacy analysis by time since stroke was a prospective analysis using 48 months as a cut-off. Treatment differences and 95% CIs for MAS, CGI, and GAS scores in patients treated ≤ 24 versus > 24 months since stroke were also analyzed and depicted using forest plots.

## Results

### Patient disposition and baseline demographics

Of 564 patients screened, 468 (83.0%; onabotulinumtoxinA, *n* = 233; placebo, *n* = 235) were randomized and constituted the ITT population (Supplementary Fig. 1). The safety population included 464 randomized patients (99.1%; onabotulinumtoxinA, *n* = 231; placebo, *n* = 233) who received ≥ 1 treatment. Those who discontinued before receiving study treatment (personal reasons, *n* = 2; protocol violation, *n* = 1; other, *n* = 1) were excluded from the safety analyses. A total of 18 patients (onabotulinumtoxinA, *n* = 10; placebo, *n* = 8) discontinued from the study during the double-blind phase, and 450 patients (onabotulinumtoxinA, *n* = 223; placebo, *n* = 227) completed the double-blind phase. No patients discontinued because of lack of efficacy.

The ITT population consisted of 153 patients (onabotulinumtoxinA, *n* = 80; placebo, *n* = 73) who were treated ≤ 24 months since stroke and 315 patients (onabotulinumtoxinA, *n* = 153; placebo, *n* = 162) who were treated > 24 months since stroke. Baseline demographics and disease characteristics including stroke severity and distribution of affected limbs were similar among patients treated ≤ 24 or > 24 months since stroke (Table 2). Compared with patients treated ≤ 24 months since stroke, patients treated > 24 months since stroke were approximately 2 years older and were slightly heavier.

**Table 2:** Baseline demographics and disease characteristics

	Time since stroke, ≤ 24 months		Time since stroke, > 24 months	
	OnabotulinumtoxinA (*n* = 80)	Placebo (*n* = 73)	OnabotulinumtoxinA (*n* = 153)	Placebo (*n* = 162)
Mean (SD) age, years	54.3 (11.9)	55.7 (12.0)	56.9 (12.9)	57.5 (11.8)
Male, *n* (%)	55 (68.8)	52 (71.2)	93 (60.8)	103 (63.6)
Caucasian, *n* (%)	68 (85.0)	62 (84.9)	116 (75.8)	132 (81.5)
Mean (SD) weight, kg	76.5 (14.8)	77.9 (14.4)	82.1 (18.9)	80.3 (15.9)
Mean (SD) height, cm	169.0 (8.0)	168.9 (9.1)	169.9 (9.6)	170.2 (9.2)
Time since stroke, years				
Mean (SD)	1.1 (0.5)	1.1 (0.5)	8.0 (6.5)	7.0 (6.7)
Median (range)	0.9 (0.4–2.0)	1.0 (0.4–2.0)	6.0 (2.1–38.4)	4.8 (2.1–54.3)
Stroke severity, *n* (%)^a^				
Mild	7 (8.8)	10 (13.7)	16 (10.5)	15 (9.3)
Moderate	55 (68.8)	42 (57.5)	105 (68.6)	108 (66.7)
Severe	18 (22.5)	21 (28.8)	32 (20.9)	39 (24.1)
Limbs affected by spasticity, *n* (%)				
Right leg only	4 (5.0)	3 (4.1)	9 (5.9)	12 (7.4)
Left leg only	9 (11.3)	9 (12.3)	14 (9.2)	15 (9.3)
Right arm and right leg	34 (42.5)	29 (39.7)	62 (40.5)	63 (38.9)
Left arm and left leg	33 (41.3)	32 (43.8)	68 (44.4)	70 (43.2)
Other	0	0	0	2 (1.2)

*SD* standard deviation

^a^Severity scores defined as mild: minor deficit, functionally non-impairing; moderate: moderate deficit, significantly interfering with activities of daily living; severe: dependent, requiring chronic care

### Efficacy endpoints

#### Overall ITT population

**Fig. 2:**
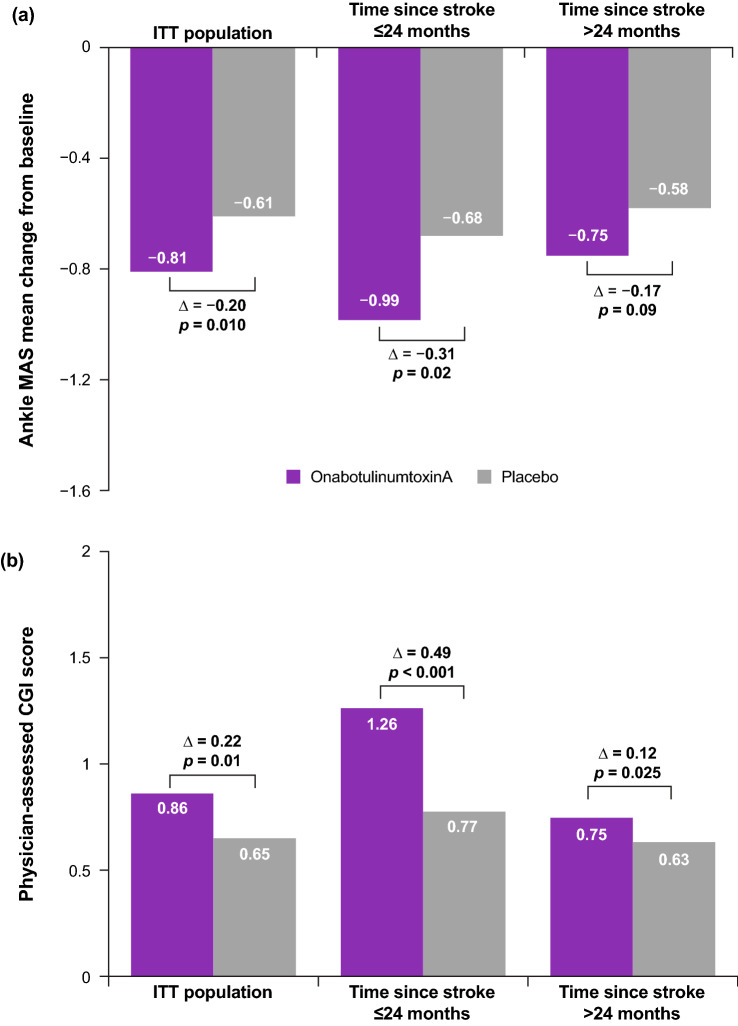
**a** Ankle MAS change from baseline and **b** physician-assessed CGI average score of weeks 4 and 6. Data are least squares means of weeks 4 and 6 change from baseline. *P* values for between-group comparisons were obtained from analysis of covariance using imputation for missing values. *CGI* clinical global impression of change, *ITT* intent-to-treat, *MAS* modified Ashworth Scale

In the overall ITT population, significantly greater improvements in MAS change from baseline (average of weeks 4 and 6) were observed with onabotulinumtoxinA (− 0.81) than with placebo (− 0.61; *p* = 0.01; Fig. 2a). Similar results were observed for the physician-assessed CGI (Fig. 2b). Main efficacy results have been published (Wein et al. 2018).

#### Efficacy based on time since stroke

Significant improvements were observed for the MAS change from baseline (average of weeks 4 and 6; Fig. 2a) among patients treated ≤ 24 months since stroke versus placebo (onabotulinumtoxinA, − 0.99; placebo, − 0.68; mean difference, − 0.31; *p* = 0.02) and those treated > 24 months since stroke (onabotulinumtoxinA, − 0.75; placebo, − 0.58; mean difference, − 0.17; *p* = 0.09). This represents a mean difference improvement of − 0.14 for the subgroup treated earlier compared with those treated later.

Significantly greater clinical improvements versus placebo on the CGI by physician (average of weeks 4 and 6; Fig. 2b) were also observed among patients treated ≤ 24 months since stroke (onabotulinumtoxinA, 1.26; placebo, 0.77; mean difference, 0.49; *p* < 0.001). In contrast, improvements were not observed in those treated > 24 months since stroke (onabotulinumtoxinA, 0.75; placebo, 0.63; mean difference, 0.12; *p* = 0.25), representing nearly a doubling in the mean difference, and thus greater improvement, in the subgroup treated earlier.

**Fig. 3:**
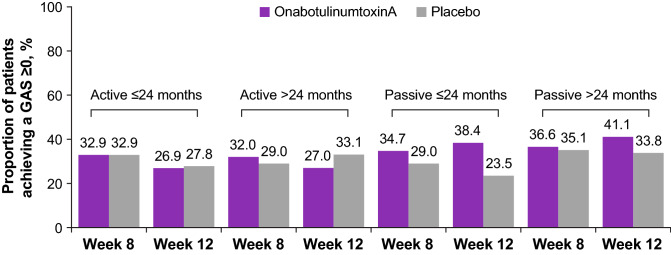
Proportion of patients achieving Goal Attainment Scale (GAS) score ≥ 0

**Fig. 4:**
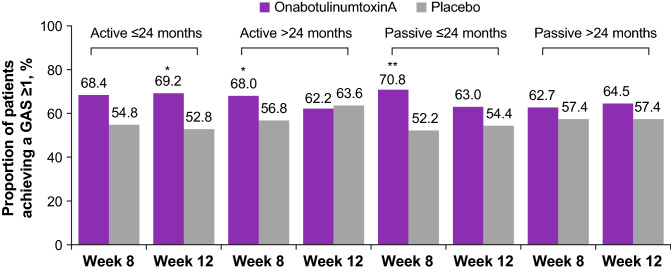
Proportion of patients achieving Goal Attainment Scale (GAS) score ≥  − 1. *P* values for between-group comparisons are determined by the Pearson Chi-square test or Fisher exact test (if > 25% of the expected cell counts are < 5). *p = 0.04; **p = 0.02

**Fig. 5:**
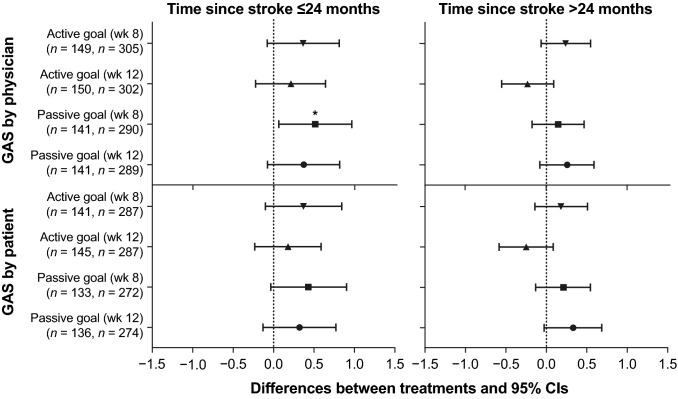
Treatment differences and 95% CIs in Goal Attainment Scale (GAS) at weeks 8 and 12 stratified by time since stroke (intent-to-treat population). Positive GAS values indicate improvement compared with baseline. *CI* confidence interval

When stratified by time of treatment initiation post-stroke, patients who were treated earlier (≤ 24 months since stroke) experienced greater improvements (mean difference from baseline vs placebo) in passive GAS scores (week 12, 0.37 vs 0.26). The responder rates for goal achievement (GAS ≥ 0) are shown in Fig. 3. Moreover, for patients treated earlier, a numerically higher rate of passive goal achievement by physician was observed in patients who received onabotulinumtoxinA treatment versus placebo at weeks 8 (onabotulinumtoxinA, 34.7%; placebo, 29.0%) and 12 (onabotulinumtoxinA, 38.4%; placebo, 23.5%). In contrast, patients who were treated later (> 24 months since stroke) showed no difference in improvement of the passive goal at week 8 for onabotulinumtoxinA (36.6%) versus placebo (35.1%). At week 12, these patients presented a marginal but insignificant increase in the proportion achieving their passive goals for onabotulinumtoxinA (41.1%) versus placebo (33.8%). The proportions of patients in each group progressing toward goal achievement (GAS ≥ − 1) are shown in Fig. 4. When stratified by time since stroke (indicating earlier vs later treatment), a significantly greater proportion of patients who received earlier onabotulinumtoxinA treatment progressed toward the physician-assessed active goal at week 12 (onabotulinumtoxinA, 69.2%; placebo, 52.8%; *p* = 0.04) and the passive goal at week 8 (onabotulinumtoxinA, 70.8%; placebo, 52.2%; *p* = 0.02). By comparison, among patients who were treated later, a significantly greater proportion of patients who received onabotulinumtoxinA progressed toward their physician-assessed active goal at week 8 (onabotulinumtoxinA, 68.0%; placebo, 56.8%; *p* = 0.04) but not at week 12 (onabotulinumtoxinA, 62.2%; placebo, 63.6%, *p* = not significant). Forest plot treatment differences for GAS scores by patient and by physician for active and passive goals at weeks 8 and 12 are stratified by treatment initiation (time since stroke; Fig. 5).

Among patients who were treated earlier, the least squares mean change from baseline in speed of gait at week 12 was − 4.0 s for onabotulinumtoxinA and − 2.1 s for placebo. For patients treated later, the mean change from baseline at week 6 was − 2.8 s for onabotulinumtoxinA and − 3.8 s for placebo. Neither comparison was statistically significant. Patient improvement, indicated by the mean difference between onabotulinumtoxinA and placebo, was greater in the earlier subgroup than in the later subgroup (− 1.9 vs − 1.0 s, respectively).

### Safety

No new safety signals were identified. In the double-blind phase of the trial, treatment-emergent AEs were reported in 41.1% (95/231) of patients treated with onabotulinumtoxinA and 34.3% (80/233) of patients treated with placebo. The majority of AEs were mild or moderate and deemed unrelated to study treatment. A detailed summary of safety has been published (Wein et al. 2018).

## Discussion

In the ITT population, onabotulinumtoxinA was safe and effective in improving MAS, CGI, and GAS scores in patients with PSLLS. This analysis further evaluated the effect of timing of onabotulinumtoxinA initiation among patients who were treated later (> 24 months since stroke) and among those who initiated treatment with onabotulinumtoxinA earlier (≤ 24 months since stroke). We observed that among those treated earlier, MAS and CGI scores improved and further progress toward active and passive goals was achieved.

The results of this analysis are similar to trends observed in previous studies of spasticity. A small (*N* = 18) pilot study of onabotulinumtoxinA in subacute (time since stroke, 4 weeks to 6 months) and chronic (time since stroke, > 5 years) stroke patients with spasticity of the hemiplegic elbow or wrist joint found that MAS score improved significantly in the elbow and wrist extensors of patients with subacute stroke but not in those with chronic stroke. Manual muscle testing of the elbow extensor, range of motion in the wrist, and modified Barthel index also improved only in the subacute group (Lim et al. 2016). Similarly, a small (*N* = 23) study in patients with lower limb spasticity associated with chronic hemiparesis found that treatment with botulinum toxin improved ankle spasticity; however, the treatment was less effective in patients with spasticity of > 1-year duration compared with those with spasticity of a shorter duration (Burbaud et al. 1996). More recently, in the BOTOX Economic Spasticity Trial, patients with upper and lower limb spasticity demonstrated a significant correlation between active functional goal achievement and time since stroke and were more likely to achieve their goals when the duration since stroke was shorter (Ward et al. 2014).

Coupled with previous findings, results of the current analysis suggest that earlier treatment of PSS with onabotulinumtoxinA may be clinically advantageous. Primary results from the double-blind phase of the REFLEX Study demonstrated improvements in ankle spasticity, as measured by MAS, CGI, and GAS (Wein et al. 2018). Improvements in MAS and CGI were observed as early as 4 weeks after the first treatment with onabotulinumtoxinA. Subsequent injections over an extended open-label treatment period have demonstrated further improvements in these scores and offer additional benefit to patients. These findings could potentially be used in the clinical setting to help clinicians and patients set expectations regarding the outcomes of treatment with onabotulinumtoxinA for PSS.

The reasons underlying the differential responsiveness to onabotulinumtoxinA of patients treated ≤ 24 months since stroke compared with those treated > 24 months since stroke, which may include prevention of secondary maladaptation and functional impairment associated with long-term spasticity, are consistent with the published literature suggesting that early treatment of spasticity may be critical for the preservation of muscle reactivity (O'Brien 2002; Wissel et al. 2009). Treatment may help prevent secondary complications and provide functionally relevant improvement in activities of daily living (Wissel et al. 2010).

A possible explanation for the observed findings is that patients with more long-standing spasticity may have more advanced structural changes in their muscles that might make them less responsive to treatment. Structural and mechanical changes described in spastic muscle include alterations in the size and type distribution of muscle fibers, proliferation of extracellular matrix material, increased spastic muscle cell stiffness (also observed in the tissue, although to a lesser degree), and compromised extracellular mechanical properties (Lieber et al. 2004). Further, Hufschmidt and Mauritz demonstrated that long-standing spasticity (≥ 1 year in duration) was associated with changes in the mechanical properties of lower leg muscles (elevated elastic resistance and increased energy consumed by ankle extensor and flexor muscles during one stretch cycle) due to gradual structural and degenerative changes leading to contracture that were not evident in patients with spasticity < 1 year in duration (Hufschmidt and Mauritz 1985).

OnabotulinumtoxinA has been shown to have a greater effect in patients with PSLLS with residual voluntary motor control and some degree of active movement than in those without voluntary motor control or whose voluntary movements were initially restricted (Kerzoncuf et al. 2015). Our results suggest that early intervention may lead to improved outcomes in patients with PSLLS and may also help prevent the development of “learned nonuse,” by which patients develop maladaptive habits post-stroke that may decrease treatment effectiveness (Esquenazi et al. 2010). Early intervention may prevent complications such as contracture tightening, which would reduce the likelihood that these maladaptive habits would develop. However, more investigation is required to understand the underlying mechanism for disease duration prior to treatment having an impact on onabotulinumtoxinA outcome in patients with PSLLS.

One potential limitation of this analysis is that stratification by early and late treatment (based on ≤ 24 or > 24 months post-stroke) was introduced post hoc. Although the open-label phase of the REFLEX study showed sustained benefit of up to 1 year of treatment with onabotulinumtoxinA in patients with PSLLS (Wein et al. 2018), the current analysis focused only on the double-blind phase, during which patients received a single dose of onabotulinumtoxinA. The stratification time point per the study protocol was ≤ 48 or > 48 months, whereas we undertook a post hoc analysis based on stratification by ≤ 24 or > 24 months post-stroke. The mean baseline time since stroke was approximately 1.1 years versus 7.5 years for ≤ 24 and > 24 months post-stroke, respectively, representing two potentially very different patient populations. Thus, it is important to interpret these data with the understanding that these were relatively small patient populations that differed in terms of this characteristic, and possibly other important dimensions. Furthermore, the study was not designed to be statistically powered for the subgroup by time since stroke analysis (≤ 24 or > 24 months); therefore, we could not determine whether statistically significant differences in improvement were present between patients treated ≤ 24 months versus > 24 months after stroke. However, the observed trend towards improved outcomes for patients treated within 24 months provides a valuable clinical basis to support future studies comparing earlier versus later intervention with onabotulinumtoxinA. Despite these limitations and the subgroup with time since stroke ≤ 24 months being substantially smaller than the > 24-month group, statistically significant differences in change in ankle MAS from baseline and CGI by physician were found for the ≤ 24-month group. Stratification by 12 months since stroke was not possible because of the low number of patients who were treated within 12 months since stroke; similarly, it would be interesting to ask in future studies whether there is a difference in outcomes for patients treated within a few months post-stroke. Typically, onset of spasticity occurs within the first few months after stroke to 24 months after stroke, with low incidence of onset after this time (Kim 2001; Sunnerhagen 2016). Finally, this analysis stratified patients according to the time since stroke rather than the time since onset of spasticity. Considering that the onset of spasticity is highly variable (Ward 2012), it is possible that time since spasticity may also be clinically relevant with regard to clinical outcomes.

Notwithstanding these limitations, the early initiation of onabotulinumtoxinA treatment showed some benefit across a range of efficacy outcomes compared with later initiation of treatment. OnabotulinumtoxinA treatment, even when administered > 24 months post-stroke, still provided a clinically relevant benefit and should be considered in patients who have not had the advantage of early initiation of onabotulinumtoxinA therapy post-stroke.

## Conclusion

OnabotulinumtoxinA 300 to 400 U is safe and effective in improving ankle MAS, CGI, and GAS scores in all patients with PSLLS regardless of time since stroke. Earlier initiation of onabotulinumtoxinA (≤ 24 vs > 24 months since stroke) provided benefit to patients with some improvements in muscle tone and global functioning as measured by physician global response scale and goal attainment; and suggest that early intervention may lead to improved patient outcomes.

## Electronic supplementary material

Below is the link to the electronic supplementary material.

Supplementary file 1 (DOCX 104 kb)
