# Supplementary figures and images for: Impact of early intervention with onabotulinumtoxinA treatment in adult patients with post-stroke lower limb spasticity: results from the double-blind, placebo-controlled, phase 3 REFLEX study
Source: J Neural Transm (Vienna). 2020 Oct 27;127(12):1619–29. doi: 10.1007/s00702-020-02251-6 (PMC7666298; doi:10.1007/s00702-020-02251-6)

**Supplementary materials**

**Supplementary Fig. 1.** Study disposition


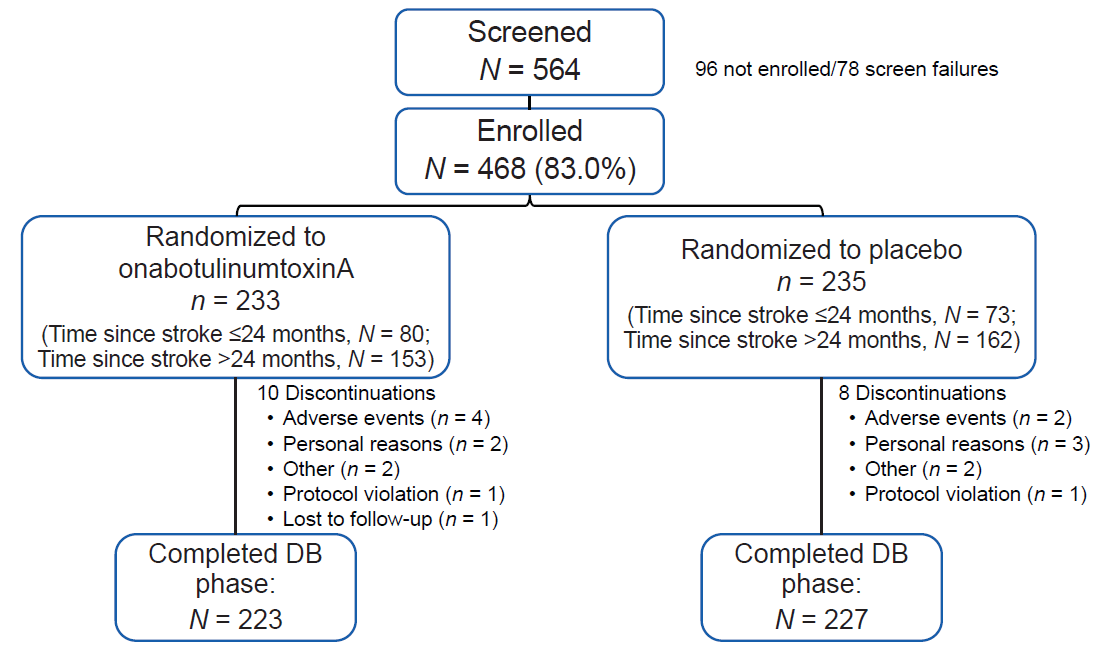

Supplement: Supplementary file 1 — (DOCX 104 kb) [file 702_2020_2251_MOESM1_ESM.docx]
